# The duration of SIRS before organ failure is a significant prognostic factor of sepsis

**DOI:** 10.1186/1865-1380-5-44

**Published:** 2012-12-31

**Authors:** Hiroki Sugita, Yoshihiro Kinoshita, Hideo Baba

**Affiliations:** 1 Department of Gastroenterological Surgery, Graduate School of, Medical Sciences, Kumamoto University, 1-1-1 Honjo, Kumamoto, 8608556, Japan; 2 Department of Aggressology and Critical Care Medicine, Graduate School of Medical Sciences, Kumamoto University, Kumamoto, Japan

**Keywords:** Severe sepsis, Septic shock, Systemic inflammatory response syndrome (SIRS), Organ failure, Acute Physiology and Chronic Health Evaluation (APACHE) II score

## Abstract

**Background:**

The mortality rate of patients complicated with sepsis-associated organ failure remains high in spite of intensive care treatment. The purpose of this study was to define the duration of systemic inflammatory response syndrome (SIRS) before organ failure (DSOF) and determine the value of DSOF as a prognostic factor in septic patients.

**Methods:**

This retrospective cohort study was conducted in an 11-bed medical and surgical intensive care unit (ICU) in a university hospital. The primary endpoint was in-hospital mortality of the septic patients.

**Results:**

One hundred ten septic patients with organ failure and/or shock were enrolled in this study. The in-hospital mortality rate was 36.9%. The median DSOF was 28.5 h. As a metric variable, DSOF was a statistically significant prognostic factor according to univariate analysis (survivor: 74.7 ± 9.6 h, non-survivor: 58.8 ± 16.5 h, *p* = 0.015). On the basis of the ROC curve, we defined an optimal cutoff of 24 h, with which we divided the patients as follows: group 1 (*n* = 50) comprised patients with a DSOF ≤24 h, and group 2 (*n* = 60) contained patients with a DSOF >24 h. There were statistically significant differences in the in-hospital mortality rate between the two groups (52.0% vs. 25.0%, *p* = 0.004). Furthermore, by multivariate analysis, DSOF ≤24 h (odds ratio: 5.89, 95% confidence interval: 1.46-23.8, *p* = 0.013) was a significant independent prognostic factor.

**Conclusion:**

DSOF may be a useful prognostic factor for severe sepsis.

## Background

The mortality rate of patients complicated with sepsis-associated organ failure or septic shock remains high in spite of the treatments in intensive care units (ICU) [1,2]. There are non-responders among the septic patients with poor prognosis whose disease progresses rapidly despite various treatments received in the ICU.

Previous reports show that certain scoring systems, including the Acute Physiology and Chronic Health Evaluation (APACHE) II score, Disseminated Intravascular Coagulation (DIC) score, Sequential Organ Failure Assessment (SOFA) score, and delta SOFA score, predict the prognosis of sepsis [3-5]. However, they sometimes fail to provide a prognosis for identifying high-risk patients with sepsis at an early stage [6,7].

Sepsis is generally defined as systemic inflammatory response syndrome (SIRS) with infection [8]. In most cases of sepsis, patients only develop SIRS in the early stage, but some of them (approximately 25%) progress to advanced stages of the disease, which entail organ failure and septic shock [9,10]. However, no study has sufficiently examined the speed of progression from early stage sepsis to organ failure and septic shock in the advanced stages. We defined a novel parameter—the duration of SIRS before organ failure (DSOF) —which denotes the speed of sepsis progression. The purpose of this study was to determine whether DSOF has prognostic value in septic patients complicated with organ failure or shock.

## Methods

### Patients and definition

This retrospective cohort study was conducted in the medical and surgical ICU of a university hospital. The institutional ethics committee approved this study, and all enrollees gave their informed consent. From September 2001 to February 2007, 118 patients were enrolled. They consist of the patients from the general ward of our hospital and patients from other hospitals. They were transferred to the ICU for treatment of sepsis.

Sepsis was defined as an infection with SIRS. Patients were classified according to the American College of Chest Physicians/Society of Critical Care Medicine Sepsis Consensus Conference, 1992 [11].

We defined SIRS as the presence of at least two of the following criteria: (1) body temperature > 38°C or < 36°C, (2) heart rate > 90 beats/min, (3) respiratory rate > 20 breaths/min or PaCo_2_ < 32 mmHg, and (4) WBC count > 12,000/μl or < 4,000/μl [8]. When patients fulfilled the criteria of SIRS, the time was recorded as first recognition. Patients who needed to receive mechanical ventilation and/or had serum Cr levels above 4.0 mg/dl or total bilirubin levels above 5.0 mg/dl were considered to have respiratory failure, renal failure, or hepatic failure, respectively. The time of intubation or blood sampling was regarded as the first recognized time of organ failure. We defined shock as systolic blood pressure below 90 mmHg or the necessity of the administration > 5 μgkg^-1^ min^-1^ dopamine, dobutamine, or any dose of nor-adrenaline, even if sufficient fluids were infused. When patients fulfilled at least one of the criteria of organ failure or septic shock, the time was recorded as first recognition.

**Figure 1:**
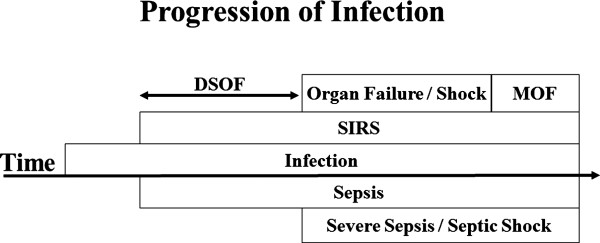
**Schematic representation of the progressive course of infection.** Sepsis without organ failure or shock in the early stage progresses to severe sepsis or septic shock in the advanced stage. Sepsis was defined as an infection with systemic inflammatory response syndrome (SIRS). Severe sepsis and septic shock were defined as sepsis with any organ failure and sepsis with shock, respectively. Duration of SIRS before organ failure (DSOF) was defined as the duration from the first recognition of SIRS to the first recognition of organ failure or septic shock.

Any patient who was diagnosed with sepsis and admitted to the 11-bed medical and surgical ICU of Kumamoto University Hospital was enrolled in this study if he or she fulfilled the criteria for sepsis with organ failure or sepsis with shock. DSOF was defined as the duration from the initial recognition of SIRS to the first recognition of organ failure or shock (Figure 1) and was calculated on the basis of accurate medical records. Various data were recorded on recognition of organ failure or shock: gender; age; shock or not shock; underlying diseases; localization of the primary infection; whether the patient came from the general ward in our hospital or from another hospital; whether the patient was post-surgical; blood pressure; heart rate; respiratory rate; bacterial species; arterial blood gas, including pH and PaO_2_; and venous blood parameters. Neurological evaluations were performed using the Glasgow Coma Scale (GCS). APACHE II and SOFA scores were calculated as described [5,12]. We defined DIC per the revised criteria of the Japanese Association for Acute Medicine (JAAM) [13,14]. Various prognostic factors were evaluated for the endpoint of in-hospital mortality of the septic patients.

As the treatment of sepsis, removal or drainage of the infection sites and adequate antimicrobial therapy were performed as soon as possible [3,15-17]. Mechanical ventilation for sepsis-induced acute lung injury (ALI)/acute respiratory distress syndrome (ARDS) and fluid resuscitation and the administration of catecholamine for maintaining circulation for septic shock are performed in septic patients to prevent the development of a more critical condition resulting in MOF and death [8,18-20]. The treatment of sepsis was performed according to the Surviving Sepsis Campaign guideline [8,21].

### Statistical analysis

The relationship between risk factors and death was first examined by univariate analysis using the two-sample unpaired Student’s *t*-test for parametric continuous variables, Mann–Whitney *U*-test for nonparametric continuous variables, and Pearson’s chi-square test for categorical variables. Multivariate logistic regression was performed to determine independent risk factors for in-hospital mortality. Risk factors with *p*-values < 0.2 by univariate analysis were put into the multivariate logistic regression. The SOFA score was not included in the multivariate analysis because it has a relationship with APACHE II scores. We analyzed the survival between patients with DSOF ≤24 h and DSOF >24 h by using Kaplan-Meier survival curves, and the significance was tested by log-rank test. All statistical analyses were performed using SPSS for Windows (Release 13.0; SPSS Inc., Chicago, IL). Two-tailed *p*-values <0.05 were considered significant.

## Results and discussion

### Results

Of the 118 patients who were enrolled initially, we excluded 5 patients who had uncertain records regarding the first recognition of SIRS and 3 patients younger than 15 years. Thus, the final group for evaluation comprised 110 patients. The mean age of the patients (72 men and 38 women) was 63.3 ± 1.4 years (range 16–88 years).

There were 59 (53.6%), 79 (71.8%), and 28 (25.5%) patients with shock, organ failure, and both, respectively. The types of organ failure were respiratory failure (70 patients), renal failure (8 patients), and hepatic failure (3 patients). The mean ICU and mean hospital stays were 15.5 ± 2.4 days and 54.1 ± 5.0 days, respectively. The mean APACHE II and SOFA scores at admission were 22.6 ± 0.7 and 8.92 ± 0.38, respectively. The mean and median DSOFs were 68.9 ± 8.5 h and 28.5 h (range 0–456 h), respectively. The in-hospital mortality rate was 36.9%.

Table 1 shows the univariate analysis of various factors and variables as prognostic factors. As a metric variable, DSOF was a statistically significant prognostic factor, as were age, APACHE II score, SOFA score, AT III, BUN, and pneumonia.

**Table 1:** Univariate analysis of risk factors for in-hospital mortality

**Prognostic factor**	**Non-survivors (*n*** **= 41)**	**Survivors (*n*** **= 69)**	**P value**
Gender (men)	30 (73.2)	42 (60.9)	0.190
Age	69.3 ± 1.7	59.7 ± 1.8	**0.001**
APACHE II score	26.6 ± 0.1	20.3 ± 0.8	**< 0.001**
SOFA score	10.1 ± 0.7	8.3 ± 0.4	**0.040**
DIC score	4.5 ± 0.32	4.37 ± 0.25	0.776
Shock	24 (58.5)	35 (50.7)	0.427
Underlying disease	27 (58.5)	37 (53.6)	0.209
Chronic cardiac insufficiency	3 (11.1)	3 (7.9)	
Diabetes mellitus	4 (14.8)	9 (23.7)	
Hepatic cirrhosis	6 (22.2)	6 (15.8)	
Immunosuppression	8 (29.6)	10 (26.3)	
Neoplasm	4 (14.8)	5 (13.2)	
Chronic renal insufficiency	1 (3.7)	1 (2.6)	
Chronic pulmonary insufficiency	1 (3.7)	3 (7.9)	
Localization of infection			
Abdomen	16 (39.0)	34 (49.3)	
Lung	19 (46.3)	19 (27.5)	**0.045**
Soft tissue	5 (12.2)	10 (14.5)	
Others	1 (2.4)	6 (8.7)	
From the general ward in our hospital	22 (53.7)	37 (53.6)	0.997
Post surgery	8 (19.5)	21 (30.4)	0.209
First antibiotic sensitivity	14 / 27 (51.9)	22 / 50 (44.0)	0.510
Time lag between first detection of SIRS and administration of antibiotics	12.8 ± 3.5	21.5 ± 6.4	0.125
GCS	11.0 ± 0.6	11.6 ± 0.4	0.432
BUN (mg/dl)	43.3 ± 4.2	32.7 ± 2.7	**0.012**
Cr (mg/dl)	2.12 ± 0.21	1.64 ± 0.17	0.051
T-bilirubin (mg/dl)	3.44 ± 0.77	2.70 ± 0.57	0.175
AT III (%)	47.6 ± 3.1	63.1 ± 2.7	**0.001**
Fibrinogen (mg/dl)	412 ± 33	463 ± 18	0.295
Platelets (×10 ^4^/μl)	11.6 ± 1.5	13.6 ± 1.6	0.619
CRP (mg/dl)	17.4 ± 1.4	16.6 ± 1.1	0.625
Ht (%)	31.0 ± 1.2	29.6 ± 0.7	0.422
DSOF (h)	58.8 ± 16.5	74.7 ± 9.6	**0.015**
DSOF (≤ 24 h)	26 (63.4)	24 (34.8)	**0.004**
DSOF (≤ 48 h)	30 (73.2)	38 (55.1)	0.059

**Figure 2:**
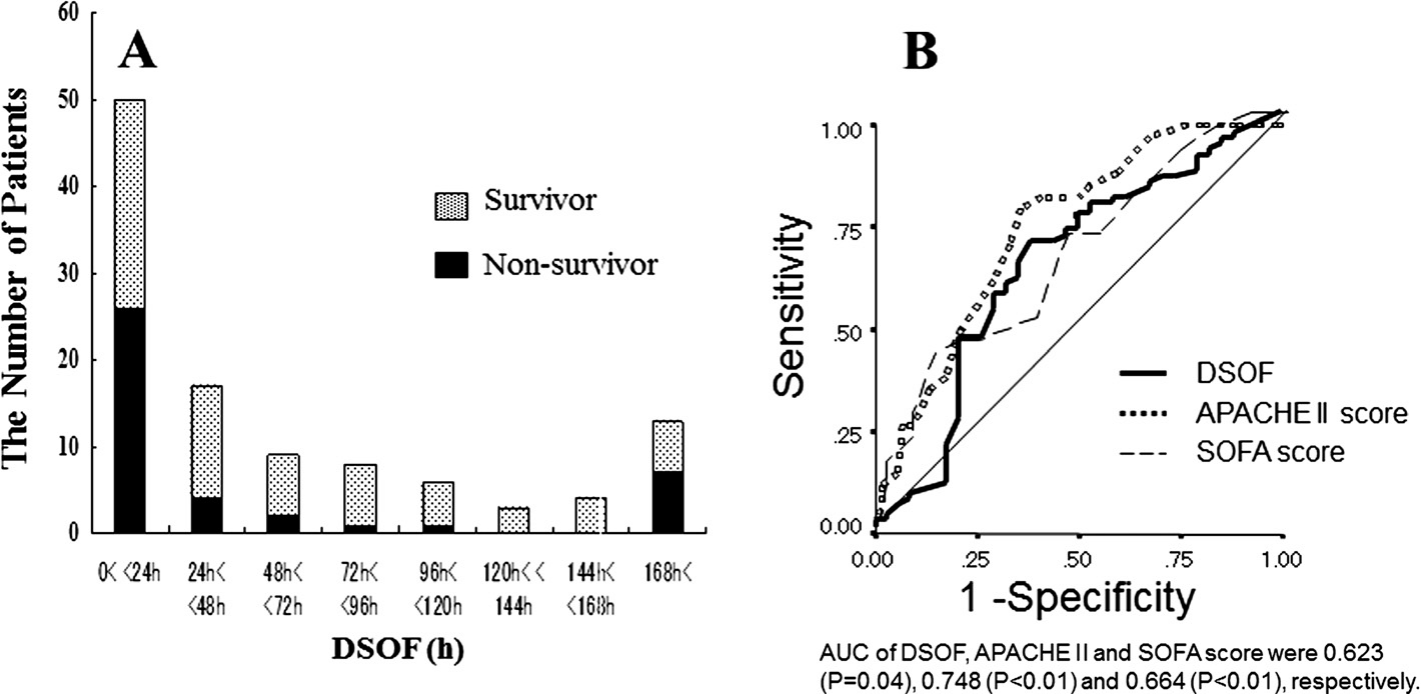
**A. The number and mortality rates of patients by DSOF. B**. ROC curve for DSOF, APACHE II, and SOFA scores for distinguishing non-survivors from patients with severe sepsis.

Figure 2A shows the number of survivors and non-survivors for each DSOF value. The mortality rate was higher in patients with shorter DSOFs. The receiver-operating characteristic (ROC) curve and area under ROC curve (AUC) show that DSOF is a significant prognostic factor as are the APACHE II and SOFA scores. On the basis of the ROC curve, we defined the DSOF cutoff as 24 h (Figure 2B). Thus, we divided the patients into two groups: group 1 consisted of patients with a DSOF of 24 h or less (≤24 h), and group 2 comprised those with a DSOF of more than 24 h (>24 h).

**Figure 3:**
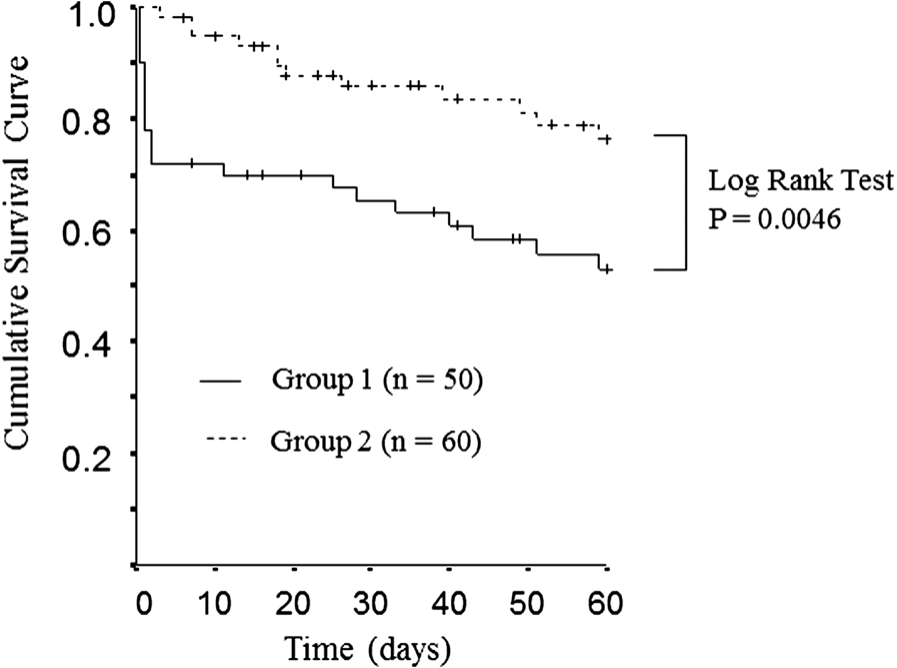
**Kaplan-Meier survival curves of septic patients with organ failure or shock.** The survival rate of the patients with DSOF ≤24 h was significantly smaller compared to that of the patients with DSOF >24 h (log rank test, *P* < 0.005). The solid line indicates group 1 (DSOF ≤24 h), and the dashed line denotes group 2 (DSOF >24 h).

In-hospital mortality rates differed significantly between the two groups (52.0% vs. 25.0%, *p* = 0.004). Figure 3 shows the Kaplan-Meier survival curves. The mortality rate in group 1 was greater than that of group 2 (log rank test, *p* <0.005).

Table 2 shows the characteristics of groups 1 and 2. The mean age in group 1 was higher than in group 2. SOFA scores and hematocrit, serum creatinine, and serum BUN levels in group 1 were significantly higher than in group 2. The incidence of septic shock was statistically greater in group 1 than in group 2.

**Table 2:** Patient characteristics in groups 1 and 2

**Characteristic**	**Group 1 (*n*** **= 50)**	**Group 2 (*n*** **= 60)**	***P *value**
Age, years	66.8 ± 1.6	60.3 ± 2.1	**0.048**
Gender (men)	32 ( 64.0 )	40 (66.7)	0.770
APACHE II score	23.3 ± 1.0	22.1 ± 0.9	0.463
SOFA score	9.88 ± 0.51	8.12 ± 0.52	**0.014**
DIC score	4.47 ± 0.28	4.38 ± 0.28	0.798
Shock	32 ( 64.0)	27 (45.0)	**0.047**
Severe underlying disease	29 (58.0)	36 (60.0)	0.832
Chronic cardiac insufficiency	1	5	0.145
Diabetes mellitus	8	5	0.215
Hepatic cirrhosis	8	4	0.118
Neoplasm	3	6	0.446
Immunosupression	6	12	0.259
Chronic renal insufficiency	2	0	0.118
Chronic pulmonary insufficiency	1	3	0.403
Localization of primary infection			
Abdomen	24 (48.0)	26 (43.3)	0.625
Lung	13 (26.0)	25 (41.7)	0.085
Soft tissue	9 (18.0)	6 (10.0)	0.223
Other	4 (8.0)	3 (5.0)	0.521
AT III (%)	56.6 ± 3.4	57.9 ± 2.9	0.643
Cr (mg/dl)	2.28 ± 0.21	1.43 ± 0.16	**< 0.001**
BUN (mg/dl)	41.9 ± 3.6	32.7 ± 3.0	**0.018**

Group 1: patients with a DSOF of 24 h or less (≤24 h); group 2: patients with a DSOF of more than 24 h (>24 h).

We analyzed the mortality rate in each subgroup on the basis of the primary infection site (Table 3). In patients with pneumonia, the in-hospital mortality rate in group 1 was significantly higher than in group 2. In the other subgroups, the in-hospital mortality rate was generally higher in group 1 than in group 2 (not significant).

**Table 3:** Mortality rate in subgroups

**Origin of infection**	**Group 1 (*n*** **= 50)**	**Group 2 (*n*** **= 60)**	***P *value**
Pneumonia	12/13 (92.3)	7/25 (28.0)	**<0.001**
Panperitonitis	5/14 (35.7)	3/7 (42.9)	0.751
Biliary tract infection	2/6 (33.3)	1/10 (10)	0.247
Necrotizing fasciitis	5/8 (62.5)	0/2 (0)	0.114
Others	3/9 (33.3)	3/16 (18.8)	

Group 1: patients with a DSOF of 24 h or less (≤24 h).

Group 2: patients with a DSOF of more than 24 h (>24 h).

In the patients infected with *E coli* or *Streptococcus* spp., the incidence was significantly greater in group 1 than in group 2. Conversely, there were significantly fewer non-survivors than survivors among the patients who were infected with *E coli*, while those with *Streptococcus* spp. tended to have high mortality rates (Table 4).

**Table 4:** Bacterial species and DSOF

**Bacteria**	**Number of patients**			**Number of patients**		
	**DSOF** ≤**24 h**	**DSOF >24 h**	***P***	**Non-survivor**	**survivor**	***P***
MRSA*	4	11		5	10	
***Streptococcus spp.***	8	2	**0.04**	7	3	0.07
***Pseudomonas aeruginosa***	2	7		5	4	
***Escherchia coli***	7	1	**0.016**	0	8	**0.02**
***Enterococcus spp.***	2	5		2	5	
***Vibrio vulnificus***	5	1	0.06	3	3	
***Klebsiella spp.***	2	4		2	4	
***Enterobacter spp.***	1	4		1	4	
***Stenotrophomonas maltophilia***	2	2		3	1	
***Haemophilus influenzae***	1	1		0	1	
***Legionella pneumophila***	0	2		0	2	
MSSA**	0	2		0	2	
***Staphylococcus epidermidis***	2	0		1	1	
***Bacillus spp.***	1	0		0	1	
***Bacteroides spp.***	0	1		0	1	
***Corynebacterium spp.***	1	0		1	0	
***Morganella morganii***	0	1		0	1	
***Mycobacterium spp.***	0	1		1	0	
***Salmonella spp.***	0	1		1	0	
***Serratia marcescens***	0	1		1	0	
***Acinetobacter spp.***	1	0		1	0	
**Fungus**	2	5		2	5	
**Others**	2	1		1	2	
**Unknown**	11	8		7	12	
**Total**	**53**	**61**		**44**	**70**	

*MRSA: methicillin-resistant *Staphylococcus aureus.*

**MSSA: methicillin-sensitive *Staphylococcus aureus.*

Various prognostic factors were analyzed by multivariate analysis using logistic regression. Table 5 shows the independent prognostic factors at the time of recognition of organ failure or shock. DSOF ≤24 h (odds ratio: 5.37, 95% confidence interval: 1.36–21.24, *p* = 0.017) was a significant prognostic factor of in-hospital mortality, as were APACHE II score, AT III levels, age, and pneumonia. As a metric variable, DSOF is not a statistically significant prognostic factor according to multivariate analysis.

**Table 5:** Multivariate analysis of risk factors for in-hospital mortality

**Risk factor**	**In-hospital mortality OR (95**% **CI)**	***P *Value**
DSOF (≤ 24 h)	5.371 (1.358-21.235)	**0.017**
Pneumonia	11.494 (2.347-55.556)	**0.003**
APACHE II score	1.189 (1.060-1.334)	**0.003**
AT III	0.956 (0.923-0.991)	**0.013**
Age	1.068 (1.014-1.125)	**0.021**
Gender (female)	0.388 (0.101-1.381)	0.153
T-bilirubin	1.030 (0.921-1.153)	0.602
BUN	0.994 (0.961-1.028)	0.717
Cr	0.911 (0.496-1.675)	0.765

### Discussion

We developed and defined the DSOF—calculated using clinical parameters—and evaluated its value as a prognostic factor (Figure 1). Our results demonstrate that as a metric variable, DSOF is a significant prognostic factor by univariate analysis and that the in-hospital mortality rate in patients with DSOF ≤24 h is significantly higher than in those with DSOF >24 h. In addition, a DSOF of ≤24 h is an independent prognostic factor in septic patients, as are APACHE II score, AT III levels, age, and primary infection in the lung according to multivariate analysis.

There may be a time lag between true SIRS development and the recognition of SIRS. The time lag may be less than a few hours. This may be a limit of this study. However, no physician can know the true development of SIRS in clinical practice. Thus, we employed the recognition time of SIRS in the present study. DSOF denotes the speed of progression from early to advanced-stage sepsis. Various factors, including host defense and its response to infection, the strength of the bacterial toxin, and treatment efficacy, influence the DSOF. Severe underlying diseases and condition such as diabetes mellitus, liver cirrhosis, and immunosuppression are implicated in the patient’s prognosis, although we did not observe any significance in this study [10,15,22-26].

Septic patients with long DSOFs might tolerate bacteria. However, the type of bacteria and toxin is implicated in the DSOF-based prognosis. For example, *Streptococcus* spp. infection caused a short DSOF and poor prognosis in this study. Thus, the DSOF might reflect the host response and the strength of the bacteria.

The wide range in DSOFs causes the difficulty in statistical evaluation. We defined an optimal cutoff of 24 h with which we divided the patients and compared mortality rates. Further, we compared the characteristics between groups 1 and 2 to determine the factors that influence the DSOF. Age might significantly influence DSOF. The higher incidence of shock in group 1 than group 2 suggests that the progression from SIRS to shock is more rapid than the progression from SIRS to organ failure. The deterioration of renal function in group 1 patients might be attributed to shock. Furthermore, the higher SOFA scores in group 1 might be attributed to renal dysfunction and shock.

We examined various prognostic factors of severe sepsis and septic shock by multivariate analysis and found that age, APACHE II score, AT III, and pneumonia are independent prognostic factors, consistent with previous reports [2,3,10,27]. Our results suggest that the DIC score at the time of ICU admission has no prognostic value, although DIC has been reported to be a prognostic factor of organ failure [14,22]. These results indicate that the progression of DIC is slower than circulatory failure and respiratory failure in many cases and that early organ failure is not dependent on DIC. We analyzed DSOFs by the primary location of infection. In septic patients in whom the primary infection was the lung, DSOF ≤24 h was a significant prognostic factor of in-hospital mortality. However, our data show that the DSOF in patients with peritonitis was shorter than that in other subgroups, and the mortality rate in patients with DSOF ≤24 h is not greater than in patients with DSOF >24 h. These results indicate that pan-peritonitis progresses more rapidly than other diseases, and the patient’s prognosis may depend on the surgical procedure and treatment after surgery. Analysis of a cohort of septic patients showing different types of sepsis may cause wide variations in DSOF. Thus, additional subgroup analyses are necessary.

DSOF may be dependent on the accuracy of the first detection of sepsis. Thus, the patients with infection should be observed carefully. However, the detection of SIRS in in-patients is not difficult. The Delta SOFA score, which measures the progression of organ failure, is an excellent prognostic factor, but it is not available until a few days after the patients have organ failure and have been admitted to the ICU [28,29]. In contrast, DSOF can be calculated at the time of recognition of organ failure. DSOF may be a promising prognostic factor for patients with severe sepsis. There is no correlation between the DSOF and APACHE II score. Thus, the combination of DSOF and APACHE II score may be more useful for determining the patients with poor prognosis.

## Conclusions

DSOF may be a promising prognostic factor for the patients with severe sepsis. Further clinical studies are necessary.

## Competing interests

The authors have no financial or other potential conflicts of interest to disclose.

## Authors’ contributions

HS thought up the design and carried out collection data, performed the statistical analysis, and drafted the manuscript. YK participated in its design and coordination. HB participated in drafting the manuscript. All authors read and approved the final manuscript.
